# Continuous facility location on graphs

**DOI:** 10.1007/s10107-021-01646-x

**Published:** 2021-03-26

**Authors:** Tim A. Hartmann, Stefan Lendl, Gerhard J. Woeginger

**Affiliations:** 1 Department of Computer Science, RWTH Aachen, Aachen, Germany; 2 Institute of Discrete Mathematics, TU Graz, Graz, Austria

**Keywords:** Covering, Location theory, Graph theory, Complexity, Parametrized complexity, 90C27, 68Q25, 05C85

## Abstract

We study a continuous facility location problem on undirected graphs where all edges have unit length and where the facilities may be positioned on the vertices as well as on interior points of the edges. The goal is to cover the entire graph with a minimum number of facilities with covering range $\delta >0$. In other words, we want to position as few facilities as possible subject to the condition that every point on every edge is at distance at most $\delta$ from one of these facilities. We investigate this covering problem in terms of the rational parameter $\delta$. We prove that the problem is polynomially solvable whenever $\delta$ is a unit fraction, and that the problem is NP-hard for all non unit fractions $\delta$. We also analyze the parametrized complexity with the solution size as parameter: The resulting problem is fixed parameter tractable for $\delta <3/2$, and it is *W*[2]-hard for $\delta \ge 3/2$.

## Introduction

We investigate the algorithmic behavior of a continuous covering problem on graphs. Consider an undirected connected graph $G=(V,E)$, whose edges are rectifiable and have unit length. Denote by *P*(*G*) the continuum set of points on all the edges and vertices. For two points $p,q\in P(G)$, denote by *d*(*p*, *q*) the length of a shortest path connecting *p* and *q* in the underlying metric space. Point *p* is said to $\delta$*-cover* point *q* for some positive real number $\delta$, if $d(p,q)\le \delta$ holds. A subset $S\subseteq P(G)$ is a $\delta$*-cover* for *G*, if for every point $p\in P(G)$ there exists some $s\in S$ that $\delta$-covers *p*. The objective is to compute for a given graph $G=(V,E)$ and for a given positive real number $\delta$ a minimum cardinality $\delta$-cover $S\subseteq P(G)$. Such a minimizing set *S* is called an *optimal* $\delta$-cover, and the cardinality |*S*| is called the $\delta$*-covering number* $\delta -{Cover}(G)$ of graph *G*.

Known and related results The area of continuous facility location on graphs has been started by Megiddo & Tamir [11], who showed that computing an optimal $\delta$-cover is NP-hard in case $\delta =2$. Furthermore [11] contains a fast algorithm for the computation of the $\delta$-covering number on trees. Kariv & Hakimi [10] establish many hardness results for discrete types of facility location problems on graphs, in which the facilities must be located on vertices. Fekete, Mitchell & Beurer [6] discuss a number of continuous facility location problems in a purely geometric setting in the Euclidean plane. We refer to the books [12] by Mirchandani & Francis and [4] by Drezner for comprehensive information on the area of facility location.

In a closely related line of research, Grigoriev & al. [9] study an obnoxious continuous facility location problem on graphs. The objective in [9] is not to cover, but to pack, and hence is dual to our objective: Place as many facilities as possible on the graph, subject to the condition that any two facilities have at least distance $\delta$ from each other. This packing problem is polynomially solvable, if $\delta$ is a rational number with numerator 1 or 2, and it is NP-hard for all other rational values of $\delta$. Finally, we mention the graph-theoretic model of Tamir [13], where every edge $e\in E$ of the underlying graph $G=(V,E)$ is rectifiable and has a given edge-dependent length $\ell (e)$. Tamir discusses the complexity and approximability of various location problems in this model with various objective functions.

Our results. We provide a complete picture of the complexity of computing the $\delta$-covering number for connected graphs $G=(V,E)$ and positive rational numbers $\delta$. We trace out the boundary between polynomial time solvability and NP-hardness, as well as the boundary between parametrized tractability and parametrized intractability.

With respect to computational complexity, the problem is NP-hard for most choices of $\delta$, as it is close to various NP-hard covering problems. Our NP-hardness reductions are inductively structured, and break down exactly at the unit fractions. The cases where $\delta$ is a unit fraction can be settled in polynomial time by applying techniques from matching theory. The results are as follows:

- If $\delta$ is a unit fraction (that is, if $\delta =1/c$ for some integer *c*), then the $\delta$-covering number can be computed in polynomial time.
- If $\delta$ is not a unit fraction, then computing the $\delta$-covering number is NP-complete.

The parametrized version of $\delta$-covering takes the solution size of a $\delta$-cover as parameter. The first intuition is that the problem should be easy for small values of $\delta$ and hard for large values of $\delta$. Indeed, if $\delta$ is small (say $\delta \le 1/4$), then $\delta$-covering essentially boils down to covering all the edges of the input graph with the facilities; this has the flavor of the VERTEX COVER problem, which is known to be fixed parameter tractable. On the other hand, if $\delta$ is large (say $\delta \ge 4$), then the main goal should be to cover all the vertices of the input graph with the facilities, whereas the edges only play a minor role and will be covered without much additional effort. Hence these cases have the flavor of the DOMINATING SET problem, which belongs to the intractable problems in the parametrized world. This intuition turns out to be correct, and we will show that at the threshold $\delta =3/2$ the parametrized complexity jumps from tractable to intractable:

- In the range $0<\delta <3/2$, the $\delta$-covering number is fixed parameter tractable.
- In the range $\delta \ge 3/2$, the $\delta$-covering number is *W*[2]-complete.

We stress that this transition occurs surprisingly sudden, and that there is no intermediate range of $\delta$ values for which this $\delta$-covering problem is *W*[1]-complete (assuming $\text {FPT}\ne W[1]$ and $W[1]\ne W[2]$ as usual).

The paper is organized as follows. Section 2 states the basic notations and presents some technical observations. Section 3 establishes a fundamental and useful connection between the $\delta$-covering number and the $\delta '$-covering number of a graph *G*, for certain values $\delta$ and $\delta '$. Section 4 gives the *W*[2]-hardness results, and Section 5 gives the NP-hardness results. The polynomial time algorithm in Section 6 for the case $\delta =1$ is mainly based on tools from matching theory. Section 7 contains one of our technical main results, an fpt-algorithm for the parametrized cases with $\delta <3/2$.

## Notation and technical preliminaries

All the graphs in this paper are undirected and connected, and all the edges have unit length. We use the word *vertex* in the graph-theoretic sense, and we use the word *point* to denote the elements of the geometric structure *P*(*G*). For a graph $G=(V,E)$ and a vertex $v\in V$, we denote by $\Gamma (v)$ the set of neighbors of *v* and we denote $\Gamma ^+(v)=\Gamma (v)\cup \{v\}$. For $V'\subseteq V$, we denote by $G[V']$ the subgraph induced by $V'$. A subset $C\subseteq V$ forms a *vertex cover* for the graph $G=(V,E)$, if every edge in *E* has at least one of its endpoints in *C*; the size of the smallest vertex cover in *G* is denoted by $\tau (G)$. A subset $M\subseteq E$ forms a *matching* for $G=(V,E)$, if no two edges in *M* share a common endpoint; the size of the largest matching in *G* is denoted by $\nu (G)$.

The *closed ball* $B^+(v,r)$ of radius *r* around point *v* contains all points $p\in P(G)$ with $d(v,p)\le r$, and the corresponding *open ball* $B^-(v,r)$ contains all *p* with $d(v,p)<r$. For an edge $e=\{u,v\}$ and a real number $\lambda$ with $0\le \lambda \le 1$, we denote by $p(u,v,\lambda )$ the point on edge *e* that is at distance $\lambda$ from vertex *u*. Note that $p(u,v,0)=u$ and $p(u,v,1)=v$, and note that $p(u,v,\lambda )=p(v,u,1-\lambda )$. For integers $\ell$ and *k*, the rational number $k/\ell$ is called $\ell$*-simple*. A set $S\subseteq P(G)$ is $\ell$-simple, if for every point $p(u,v,\lambda )$ in *S* the number $\lambda$ is $\ell$-simple.

### Lemma 2.1

Let $c\ge 1$ be an integer, let *G* be a graph, and let $G'$ be the graph that results from *G* by subdividing every edge into *c* new edges. Then $\delta -{Cover}(G)=(c\cdot \delta )-{Cover}(G')$.

### Proof

As the subdivision stretches the metric space *P*(*G*) by a factor *c*, the $\delta$-covers in *G* are in one-to-one correspondence with the $(c\cdot \delta )$-covers in $G'$. $\square$

### Lemma 2.2

Let $G=(V,E)$ be a graph and let $\delta =a/b$ with integers *a* and *b*. Then there exists an optimal $\delta$-cover $S^*$ that is 2*b*-simple.

### Proof

We start with the cases where $b=1$ and where $\delta$ is integer. Let *S* be an optimal $\delta$-cover for graph *G*, and replace every point $p=p(u,v,\lambda )$ with $0\le \lambda \le 1/2$ in *S* by a corresponding point $p^*$: If $\lambda =0$ then $p^*=u$, and if $0<\lambda \le 1/2$ then $p^*=p(u,v,1/2)$. Since all points in the resulting set $S^*$ are either vertices or midpoints of edges, the set is 2-simple. We claim that set $S^*$ is also a $\delta$-cover. Indeed, the points on any edge $\{x,y\}\in E$ are covered by at most two points $s_x,s_y\in S$; in other words, there exists a real number $\mu$ with $0\le \mu \le 1$ and there exist two points $s_x,s_y\in S$ so that $s_x$ covers the points $p(x,y,\lambda )$ with $0\le \lambda \le \mu$ and so that $s_y$ covers the points $p(x,y,\lambda )$ with $\mu \le \lambda \le 1$. Then the two points $s^*_x$ and $s^*_y$ in $S^*$ together cover the entire edge $\{x,y\}$. All in all, $S^*$ is an optimal $\delta$-cover and 2-simple, exactly as desired.

In the cases where $\delta =a/b$ for some $b\ge 2$, we apply Lemma 2.1 with a stretching factor of *b*. The above discussion and Lemma 2.1 yield an optimal $\delta$-cover that is 2*b*-simple. $\square$

### Lemma 2.3

For every fixed rational $\delta$, the $\delta$-covering problem is contained in NP.

### Proof

This is an easy consequence of Lemma 2.2. We take an optimal 2*b*-simple $\delta$-cover $S^*$ as NP-certificate. Then 2*b*-simplicity ensures a polynomially bounded encoding length of the certificate, while verifying the certificate in polynomial time is straightforward. $\square$

## The shifting theorem

This section is dedicated to the proof of the following theorem, which we call the *“shifting theorem”*. This theorem allows us to shift certain statements on the value $\delta -{Cover}(G)$ to closely related statements on the value $\delta '-{Cover}(G)$, for certain combinations of the values $\delta$ and $\delta '$. The proof is technical and long, and needs a number of tedious case distinctions. We remark that in the rest of the paper, only the statement of the theorem itself will be used, but no insights or details from the proof.

### Theorem 3.1

Let $G=(V,E)$ be a graph, and let *a* and *b* be positive integers. Then

1 $$\begin{aligned} \frac{a}{2a+b}-{Cover}(G) = \frac{a}{b}-{Cover}(G) + |E|. \end{aligned}$$

Throughout the proof, we will denote $\delta =a/b$ and $\delta '=a/(2a+b)$. Note that $\delta '<1/2$, and note that hence every $\delta '$-cover must contain an interior point from every edge $e\in E$.

### The first half of the proof

The first half of the proof establishes that in equation (1) the left hand side is at most the right hand side. Hence let $S\subseteq P(G)$ be an optimal $\delta$-cover for *G*. We construct a new set $S'\subseteq P(G)$ from *S* by considering two cases for every edge $e=\{u,v\}\in E$:

(i) If edge $e=\{u,v\}$ contains at least one point from the cover *S*, we let $p(u,v,\lambda _i)$ with $1\le i\le k$ denote these points in *S* on *e*, where $0\le \lambda _1<\cdots <\lambda _k\le 1$. Without loss of generality we assume that $\lambda _{i+1}-\lambda _i=2\delta$ for $1\le i\le k-1$; this in particular yields $\lambda _k=\lambda _1+2(k-1)\delta$. We define $k+1$ real numbers $\mu _0,\mu _1,\ldots ,\mu _k$ with $\mu _0=\lambda _1 b/(2a+b)$ and with $\mu _{i+1}-\mu _i=2\delta '$ for $0\le i\le k-1$. Note that this yields $\mu _k=(2a+\lambda _k b)/(2a+b)$, and that furthermore $0\le \mu _0<\mu _1<\cdots <\mu _k\le 1$ holds. We put the corresponding $k+1$ points $p(u,v,\mu _i)$ with $0\le i\le k$ into $S'$.
(ii) If edge $e=\{u,v\}$ does not contain any point from the cover *S*, then we let $p_u$ be a point in *S* at minimum distance to *u* and we let $p_v$ be a point in *S* at minimum distance to *v*. Denote $d_u=d(u,p_u)$ and $d_v=d(v,p_v)$, and assume without loss of generality that $d_u\le d_v$. We define $\mu _e= (2a-b\,d_u)/(2a+b)$, and we note that $d_u\le \delta$ implies $0<\mu _e<1$. We put the single point $q_e=p(u,v,\mu _e)$ into $S'$.

This completes the construction of set $S'$. We point out the following hidden symmetry in the construction under (i): The distance from vertex *u* to the nearest point from *S* on the edge equals $\lambda _1$, and the distance from vertex *u* to the nearest point from $S'$ on the edge equals $\mu _0$. The ratio between these two distances is $\mu _0/\lambda _1=b/(2a+b)$. Similarly, the distance from vertex *v* to the nearest point from *S* on the edge equals $1-\lambda _k$, and the distance from vertex *v* to the nearest point from $S'$ on the edge equals $1-\mu _k$. The ratio $(1-\mu _k)/(1-\lambda _k)$ between these two distances again is $b/(2a+b)$.

#### Lemma 3.2

If edge $e=\{u,v\}$ contains some point from *S*, then it is $\delta '$-covered by $S'$.

#### Proof

Recall that edge *e* contains the *k* points $p(u,v,\lambda _i)$ with $1\le i\le k$ from *S* and the $k+1$ points $p(u,v,\mu _i)$ with $0\le i\le k$ from $S'$. Since $\mu _{i+1}-\mu _i=2\delta '$ holds for $0\le i\le k-1$, all points $p(u,v,\tau )$ with $\mu _0\le \tau \le \mu _k$ are $\delta '$-covered by $S'$. It remains to consider the points $p(u,v,\tau )$ with $0\le \tau \le \mu _0$ and with $\mu _k\le \tau \le 1$; because of symmetry we will only discuss the former case.

If $\lambda _1\le \delta$ holds, then $\mu _0=\lambda _1 b/(2a+b)\le \delta '$, so that point $p(u,v,\tau )$ is $\delta '$-covered by the point $p(u,v,\mu _0)\in S'$. In the remaining cases $\lambda _1>\delta$ holds, and we consider the point $x\in S$ that is closest to vertex *u*. As the distance between *u* and *x* is at most $\lambda _1\le 1$, point *x* lies on some other edge $\{u,w\}$ incident to vertex *u*. This yields $x=p(u,w,\xi )$ for some $\xi <\delta$. Since all points on the shortest path between *x* and $p(u,v,\lambda _1)$ are $\delta$-covered by *S*, we conclude $\lambda _1+\xi \le 2\delta$. Now by our construction (i), set $S'$ contains the point $x'=p(u,w,\xi ')$ with $\xi '=\xi b/(2a+b)$. By combining these observations, we get

$$\begin{aligned} \mu _0+\xi ' = \lambda _1 \frac{b}{2a+b}+ \xi \frac{b}{2a+b} \le 2\delta \frac{b}{2a+b} = 2\delta '. \end{aligned}$$

Hence all the points on the shortest path between $x'$ and $p(u,v,\mu _0)$, and in particular all points $p(u,v,\tau )$ with $\tau \le \mu _0$, are $\delta '$-covered by $S'$. $\square$

#### Lemma 3.3

If edge $e=\{u,v\}$ does not contain any point from *S*, then *e* is $\delta '$-covered by $S'$.

#### Proof

Recall that $p_u$ and $p_v$ are points in *S* at minimum distance to *u* and *v*, and that their corresponding distances are denoted $d_u$ and $d_v$. For $d_u\ge d_v$, the point $q_e=p(u,v,\mu _e)$ with $\mu _e= (2a-b\,d_u)/(2a+b)$ is in $S'$. As edge *e* does not contain any point from *S*, all points on *e* are $\delta$-covered by $p_u$ and $p_v$. This in particular yields $d_u\le \delta$ and $d_v\le \delta$.

We claim that there exists some point $x\in S'$ with $d(u,x)\le b\,d_u/(2a+b)$. First assume that point $p_u$ lies on an edge $\{u,w\}$ incident to *u*. Then our construction yields that this edge contains a point $p'_u\in S'$ with $d(u,p'_u)\le d_u\,b/(2a+b)$; hence in this case $x=p'_u$ is the desired point. Next assume that the point $p_u$ does not lie on an edge incident to *u*. Then consider the neighbor *w* of *u* that lies on the shortest path connecting vertex *u* to point $p_u$. Since the edge $f=\{u,w\}$ does not contain any point from *S* and since $d_w=d_u-1<d_u$ holds, our construction puts the point $q_f=p(w,u,\mu _f)$ with $\mu _f=(2a-b\,d_w)/(2a+b)$ into set $S'$. By choosing $x=q_f$, the distance *d*(*u*, *x*) equals $1-\mu _f=b\,d_u/(2a+b)$. Hence, in both cases the desired point *x* does indeed exist.

Finally we want to show that every point $p(u,v,\tau )$ on edge *e* is $\delta '$-covered by $S'$. First we discuss the cases with $0\le \tau \le \mu _e$. If $x=q_e$, then $d(u,x)\le b\,d_u/(2a+b)$ and $d_u\le \delta$ imply $\mu _e\le \delta '$; hence the point $p(u,v,\tau )$ is $\delta '$-covered by $q_e\in S'$. If $x\ne q_e$, then we observe

$$\begin{aligned} d(q_e,x) = d(q_e,u)+d(u,x) ~\le ~ \frac{2a-b\,d_u}{2a+b} + \frac{b\,d_u}{2a+b} = 2\delta '. \end{aligned}$$

Hence the point $p(u,v,\tau )$ is $\delta '$-covered by one of the points $x,q_e\in S'$. The second case discusses the remaining points $p(u,v,\tau )$ on edge *e* with $\mu _e\le \tau \le 1$. In the first subcase we assume $d_v=d_u+1$, so that *v* is $\delta$-covered by point $p_u$. Then $d_u+1=d_v\le \delta$ and $1-\mu _e=b\,(d_u+1)/(2a+b)\le \delta '$ show that point $p(u,v,\tau )$ is $\delta '$-covered by $q_e\in S'$. In the second subcase we assume $d_v<d_u+1$. Here we reuse our above argument that gave us the existence of point *x*: We show the existence of some point $y\in S'$ with $d(v,y)\le b\,d_v/(2a+b)$. Then we conclude that the point $p(u,v,\tau )$ is $\delta '$-covered either by $q_e$ or by *y*: If $y=q_e$, then $d(v,q_e)\le \delta '$, and point $p(u,v,\tau )$ is $\delta '$-covered by $q_e\in S'$. If $y\ne q_e$, then $d(q_e,y)\le 2\delta '$, and the point $p(u,v,\tau )$ is $\delta '$-covered by one of the points $y,q_e\in S'$. This resolves the last subcase of the final case, and hence completes the proof. $\square$

Lemmas 3.2 and 3.3 show that $S'$ is a $\delta '$-cover for graph *G*. Since every edge carries one more point from $S'$ than from *S*, we furthermore have $|S'|=|S|+|E|$. Hence the $\delta '$-covering number is at most $|S'|=|S|+|E|$, and the left hand side in equation (1) is less or equal to the right hand side.

### The second half of the proof

The second half of the proof establishes that in equation (1) the right hand side is less or equal to the left hand side. Hence let $S'\subseteq P(G)$ be an optimal $\delta '$-cover for *G*, and let $e=\{u,v\}$ be an edge that contains $k+1$ points from $S'$. Since $\delta '<1/2$, the interior of edge *e* contains at least one point from $S'$ so that we have $k\ge 0$. Let $p'_i=p(u,v,\mu _i)$ with $0\le i\le k$ be an enumeration of the points from $S'$ on edge *e*, where $0\le \mu _0<\mu _1<\cdots <\mu _k\le 1$. Without loss of generality we may assume that the points $p'_0,\ldots ,p'_k$ satisfy one of the following:

- $p'_0=u$ and $p'_k=v$, and $\mu _{i+1}-\mu _i\le 2\delta '$ for $0\le i\le k-1$.
- $p'_0\ne u$ or $p'_k\ne v$, and $\mu _{i+1}-\mu _i=2\delta '$ for $0\le i\le k-1$.

Note that in the cases with $k\ge 1$ we either have $\mu _0=0$ or $\mu _1-\mu _0=2\delta '$; in either case this yields the bound $\mu _0\le 1-2\delta '$. Note furthermore that $\mu _k\le \mu _0+2k\delta '$ holds, as otherwise $S'$ would not $\delta '$-cover the complete edge *e*. We construct a new set $S\subseteq P(G)$ from $S'$ as follows:

(i) If $k=0$, set *S* does not contain any point from edge *e*.
(ii) If $k\ge 1$, we define *k* real numbers $\lambda _1,\lambda _2,\ldots ,\lambda _k$ by setting $\lambda _1=\mu _0(2a+b)/b$ and $\lambda _k=((2a+b)\mu _k-2a)/b$. The remaining values are fixed by $\lambda _i-\lambda _{i-1}=2\delta$ for $2\le i\le k-1$. Note that the bound $\mu _0\le 1-2\delta '$ implies $\lambda _1\le 1$, and that $\mu _k\le 1$ implies $\lambda _k\le 1$. We put the *k* points $p_i=p(u,v,\lambda _i)$ with $1\le i\le k$ into *S*.

This completes the definition of set *S*. Similarly as in the preceding subsection, there is a hidden symmetry in construction (ii): The distance from vertex *u* to the nearest point from $S'$ on the edge equals $\mu _0$, and the distance from vertex *u* to the nearest point from *S* on the edge equals $\lambda _1$. The ratio between these two distances is $\lambda _1/\mu _0=(2a+b)/b$. The distance from vertex *v* to the nearest point from $S'$ on the edge is $1-\mu _k$, and the distance from vertex *v* to the nearest point from *S* on the edge equals $1-\lambda _k$. The ratio between these two distances again is $(1-\lambda _k)/(1-\mu _k)=(2a+b)/b$.

Clearly $|S|=|S'|-|E|$. To complete the proof of the Shifting Theorem 3.1, we will show that this set *S* constitutes a $\delta$-cover for graph *G*.

#### Lemma 3.4

If edge $e=\{u,v\}$ contains at least two points from $S'$, then *e* is $\delta$-covered by *S*.

#### Proof

A major part of the argument runs in parallel with the proof of Lemma 3.2. First we rewrite the bound $\mu _k\le \mu _0+2k\delta '$ (as observed above) into the equivalent inequality $\lambda _k\le \lambda _{k-1}+2\delta$. This shows that every point $p(u,v,\tau )$ with $\lambda _1\le \tau \le \lambda _k$ is $\delta$-covered by *S*. It remains to consider the points $p(u,v,\tau )$ with $0\le \tau \le \lambda _1$ and the points with $\lambda _k\le \tau \le 1$; because of symmetry we will only discuss the former case. If $\mu _0\le \delta '$, then $\lambda _1\le \delta$ and point $p(u,v,\tau )$ is $\delta$-covered by *S*. In the remaining cases we have $\mu _0>\delta '$.

We consider the point $x'\in S'$ that is closest to vertex *u*. Since $\delta '<1/2$, this point $x'$ lies on some edge $\{u,w\}$ incident to vertex *u*; we conclude $x'=p(u,w,\xi ')$ with $\xi '<\mu _0\le 1-2\delta '$. Since the points on the shortest path between $x'$ and $p'_1$ are $\delta '$-covered by $x'$ and $p'_1$, we get $\xi '+\mu _0\le 2\delta '$. Now consider the point $y'=p(u,w,1-\delta ')$: As the distance from $y'$ to $x'$ is $1-\delta '-\xi '>\delta$, this point is not $\delta '$-covered by $x'$. And since the distance from $y'$ to the end-vertices *u* and to *w* is at least $\delta$, the point can only be $\delta '$-covered by points on the edge $\{u,w\}$. All in all, these observations yield that the edge $\{u,w\}$ contains at least two points from $S'$. Now by construction (ii), the set *S* does contain the point $x=p(u,w,\xi )$ with $\xi =\xi '\,(2a+b)/b$. Since the inequality $\xi '+\mu _0\le 2\delta '$ implies the inequality $\xi +\lambda _1\le 2\delta$, all the points on the shortest path from *x* to $p(u,v,\lambda _1)$, and in particular all points $p(u,v,\tau )$ with $\tau \le \lambda _1$, are $\delta$-covered by *S*. $\square$

Finally, we consider an edge $e=\{u,v\}$ that contains only a single point from $S'$. We introduce an auxiliary path $P_u$ through vertices $u_0,u_1,\ldots ,u_{\ell +1}\in V$ (with $\ell \ge 0$) and an associated sequence of points $q'_0,\ldots ,q'_{\ell }\in S'$ that are inductively defined as follows: The first vertex on path $P_u$ is vertex $u_0=u$. Once vertex $u_i$ has been fixed, we pick point $q'_i$ as the point in $S'$ that is closest to $u_i$; ties are broken lexicographically. Since $\delta '<1/2$, this point $q'_i\in S'$ lies on an edge $\{u_i,u_{i+1}\}$ incident to vertex $u_i$, which leads to the next vertex $u_{i+1}$ on path $P_u$. The path ends, if an edge $\{u_i,u_{i+1}\}$ contains two or more points from $S'$; this final edge is denoted $\{u_{\ell },u_{\ell +1}\}$. To summarize, each of the first $\ell$ edges on $P_u$ contains exactly one point from $S'$, whereas the final edge $\{u_{\ell },u_{\ell +1}\}$ contains at least two points from $S'$. On every edge $\{u_i,u_{i+1}\}$, the associated point $q'_i\in S'$ is closer to $u_i$ than to $u_{i+1}$ (as its distance to $u_i$ is at most $\delta '<1/2$, while its distance to $u_{i+1}$ is at least $1-\delta '>1/2$). Note furthermore that among the vertices $u_0,u_1,\ldots ,u_{\ell +1}$ on $P_u$, only the final vertex $u_{\ell +1}$ can be in $S'$.

#### Lemma 3.5

The auxiliary path $P_u$ through the vertices $u_0,u_1,\ldots ,u_{\ell +1}$ is simple.

#### Proof

We define for every vertex $u_i$ on path $P_u$ the non-negative real number $d_i=d(u_i,q'_i)$, that is, the distance of $u_i$ to the nearest point in $S'$. We claim that these numbers are strictly decreasing along $P_u$ and satisfy $d_i>d_{i+1}$. Suppose for the sake of contradiction that $d_i\le d_{i+1}$ for some $i\ge 0$. Consider the point $x=p(u_i,u_{i+1},d_i+1/2)$; this point is well-defined since $d_i+1/2<\delta '+1/2<1$. Then point *x* is not $\delta '$-covered by $q'_i\in S'$, since $d(q'_i,x)=1/2>\delta '$. And point *x* is not $\delta '$-covered by $q'_{i+1}$, since

$$\begin{aligned} d(x,q'_{i+1})= & {} d(u_i,u_{i+1}) +d(u_{i+1},q'_{i+1}) -d(u_i,q'_i) -d(q'_i,x) \\= & {} 1+d_{i+1}-d_i-1/2 \ge 1/2 > \delta '. \end{aligned}$$

Since all other points in $S'$ are farther away from *x* than $q'_i$ and $q'_{i+1}$, we get that *x* is not $\delta '$-covered by $S'$. This contradiction establishes the claim, and the claim clearly implies the statement of the lemma. $\square$

#### Lemma 3.6

If edge $e=\{u,v\}$ contains exactly one point from $S'$, then it is $\delta$-covered by *S*.

#### Proof

Let $p_e\in S'$ be the unique point from $S'$ on *e*. Since $\delta '<1/2$, this point lies in the interior of *e*. Let $u_0,u_1,\ldots ,u_{\ell +1}\in V$ (with $\ell \ge 0$) be the vertices on the auxiliary path $P_u$, and let $q'_0,\ldots ,q'_{\ell }\in S'$ be the corresponding associated points. Let $v_0,v_1,\ldots ,v_{m+1}\in V$ (with $m\ge 0$) be the vertices on the analogously defined auxiliary path $P_v$ that starts at $v_0=v$, and let $q''_0,\ldots ,q''_m\in S'$ be the corresponding associated points. Furthermore we denote $\Delta _u=d(u_{\ell },q'_{\ell })$ and $\Delta _v=d(v_m,q''_m)$.

Let us first discuss the case where the paths $P_u$ and $P_v$ are vertex-disjoint. We consider the part *X* of *P*(*G*) that consists of path $P_u$ without its final piece between point $q'_{\ell }$ and vertex $u_{\ell +1}$, of the edge $e=\{u,v\}$, and of path $P_v$ without its final piece between point $q''_m$ and vertex $v_{m+1}$. In other words, *X* is a path-shaped piece of *P*(*G*) that starts in point $q'_{\ell }$ and ends in point $q''_m$. The total (metric) length of *X* equals $\Delta _u+\ell +1+m+\Delta _v$. The points in *X* are $\delta '$-covered by point $p_e$, by $q'_0,\ldots ,q'_{\ell }$, and by $q''_0,\ldots ,q''_m$. The two endpoints $q'_{\ell }$ and $q''_m$ of *X* each cover a piece of length $\delta '$ of *X*, while each of the other $\ell +m+1$ points covers a piece of length $2\delta '$ of *X*. This yields

2 $$\begin{aligned} (\Delta _u+\Delta _v) \,+\, (\ell +m+1) ~\le ~ 2(\ell +m+2)\delta '. \end{aligned}$$

Now by construction (ii), set *S* contains the point $x_u$ on edge $\{u_{\ell },u_{\ell +1}\}$ that is exactly at distance $\Delta _u(2a+b)/b$ from vertex $u_{\ell }$, and *S* also contains the point $x_v$ on edge $\{v_m,v_{m+1}\}$ that is exactly at distance $\Delta _v(2a+b)/b$ from vertex $v_m$. The inequality (2) can be rewritten into the equivalent inequality

3 $$\begin{aligned} (\Delta _u+\Delta _v)\frac{2a+b}{b} \,+\, (\ell +m+1) ~\le ~ 2\delta . \end{aligned}$$

The inequality (3) yields that the entire piece *X* (and hence in particular edge *e*) is $\delta$-covered by the two points $x_u\in S$ and $x_v\in S$.

It remains to discuss the cases where the two paths $P_u$ and $P_v$ are not vertex-disjoint. In the case where neither path $P_u$ nor path $P_v$ starts with edge *e*, the two paths are initially disjoint, then meet in some vertex, and from that vertex on run along the same edges. We define *X* similarly as above as the union of edge *e*, of path $P_u$ without its final piece, and of path $P_v$ without its final piece, but this time we clone and double the overlapping piece of $P_u$ and $P_v$ and the associated points on this piece. Then inequality (2) holds exactly as before, implies (3) exactly as before, and hence yields that edge *e* is $\delta$-covered by *S* exactly as before.

In the only remaining case, edge *e* is the starting edge of $P_u$ or $P_v$, say of path $P_u$. In other words, path $P_u$ consists of edge *e* and of path $P_v$, and the first associated point $q'_1$ on $P_u$ coincides with $p_e\in S'$. We define $X\subset P(G)$ as the path $P_u$ without its final piece between point $q'_{\ell }$ and vertex $u_{\ell +1}$. Now *X* is $\delta '$-covered by $q'_0,\ldots ,q'_{\ell }$, where the endpoint $q'_{\ell }$ covers a piece of length $\delta '$ and where each of the other $\ell$ points covers a piece of length $2\delta '$. Since the total (metric) length of *X* equals $\Delta _u+\ell$, this yields

4 $$\begin{aligned} \Delta _u+\ell \le (2\ell +1)\delta '. \end{aligned}$$

By construction (ii), set *S* contains the point $x_u$ on $\{u_{\ell },u_{\ell +1}\}$ that is at distance $\Delta _u(2a+b)/b$ from $u_{\ell }$. Now all of *X* (and in particular edge *e*) is $\delta$-covered by $x_u\in S$, since inequality (4) is equivalent to $\ell +\Delta _u(2a+b)/b\le \delta$. This completes the proof of the lemma. $\square$

## Parametrized hardness results

In this section we prove the following theorem.

### Theorem 4.1

For every fixed rational $\delta$ with $\delta \ge 3/2$, the $\delta$-covering problem with the solution size *k* as parameter is *W*[2]-complete.

The proof of Theorem 4.1 will be done in four steps: Lemma 4.5 settles hardness for the cases with $3/2\le \delta <2$, Lemma 4.6 settles the cases with $\ell \le \delta <\ell +1/2$ for every integer $\ell \ge 2$, and Lemma 4.7 settles the remaining cases with $\ell +1/2\le \delta <\ell +1$ for every $\ell \ge 2$. Lemma 4.8 finally establishes containment in *W*[2].

Some of our fpt-reductions are based on a variant of the dominating set problem that we call COLORFUL DOMINATING SET. Recall that a vertex *u* *dominates* a vertex *v*, if and only if $u\in \Gamma ^+(v)$. Recall furthermore that a subset *D* of vertices is a *dominating set*, if every vertex in the graph is dominated by some vertex in *D*.

Problem: COLORFUL DOMINATING SET

Instance: An undirected, connected graph $H=(V_H,E_H)$ whose vertex set $V_H$ is partitioned into *k* color classes $V_1,V_2,\ldots ,V_k$.

Question: Do there exist vertices $u_i\in V_i$ for $1\le i\le k$ that form a dominating set?

We suspect that the following lemma is already known, but we have not been able to locate it in the literature. Hence we state the (straightforward) proof.

### Lemma 4.2

COLORFUL DOMINATING SET is *W*[2]-hard.

### Proof

The fpt-reduction is done from the classical DOMINATING SET problem with solution size $k'$ as parameter, which is well-known to be *W*[2]-hard [1–3]: Given a graph $G=(V_G,E_G)$ and an integer $k'$, decide whether *G* possesses a dominating set of size $k'$. We construct the following instance $H=(V_H,E_H)$ of COLORFUL DOMINATING SET.

- For every vertex $v\in V_G$, we put *k* corresponding vertices $v^1,\ldots ,v^k$ into $V_H$. Vertex $v^i$ belongs to color class $V_i$. Furthermore, we put all edges between these *k* vertices into $E_H$.
- For every edge $\{u,v\}\in E_G$ and for all *i*, *j* with $1\le i,j\le k$, we put $\{u^i,v^j\}$ into $E_H$.

Finally we set the parameter as $k:=k'$. If *G* contains a *k*-element dominating set $\{u_1,\ldots ,u_k\}$, then the *k* corresponding vertices $u_i^i$ with $1\le i\le k$ form a colorful dominating set in *H*. Vice versa, if *H* contains a colorful dominating set of cardinality *k*, then dropping the superscripts yields a *k*-element dominating set for *G*. $\square$

Now let us turn to the $\delta$-covering number, and let us fix some rational number $\delta$ in the range $3/2\le \delta <2$. We fpt-reduce from an instance $H=(V_H,E_H)$ with color classes $V_1,V_2,\ldots ,V_k$ of COLORFUL DOMINATING SET by constructing the following instance $G=(V_G,E_G)$ of $\delta$-covering.

- The vertex set $V_G$ contains every vertex $u\in V_H$ together with two copies $u'$ and $u''$. Furthermore, there are 2*k* vertices $x_1,\ldots ,x_k$ and $y_1,\ldots ,y_k$.
- The edge set $E_G$ contains for every *i* with $1\le i\le k$ all the edges on $V_i\cup \{x_i,y_i\}$. Furthermore, $E_G$ contains the triangle on $u,u',u''$ for every $u\in V_H$. Finally, for every edge $\{u,v\}\in E_H$ the set $E_G$ contains $\{u,v\}$, the two cross-edges $\{u,v'\}$ and $\{u,v''\}$ and (by symmetry) the two cross-edges $\{v,u'\}$ and $\{v,u''\}$.

**Fig. 1:**
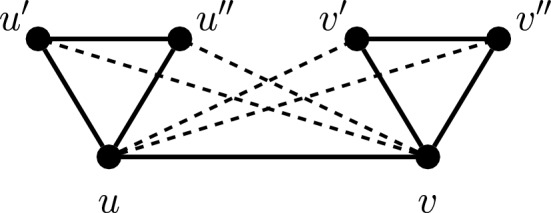
Illustration for the fpt-reduction for $3/2\le \delta <2$. The cross-edges between the two triangles $u,u',u''$ and $v,v',v''$ are shown as dashed lines

### Lemma 4.3

If graph *H* possesses a colorful dominating set, then $\delta -{Cover}(G)\le k$.

### Proof

As $V_H\subseteq V_G$, the colorful dominating set *D* in *H* induces a set $S_D$ of *k* corresponding points in *P*(*G*). We claim that this point set $S_D$ forms a $\delta$-cover for *P*(*G*).

Let us first consider some point *p* on one of the edges $\{u_1,u_2\}$ in the clique induced by $V_i\cup \{x_i,y_i\}$. As $V_i$ contains some vertex $v\in D$, point *p* is at distance at most $d(v,u_1)+d(u_1,p)$ and at distance at most $d(v,u_2)+d(u_2,p)$ from point $v\in S_D$. Since $d(v,u_1)=d(v,u_2)=1$ and since $d(u_1,p)+d(u_2,p)=1$, this means that *p* is at distance at most $3/2\le \delta$ from point $v\in S_D$.

Next, consider a point *p* on one of the edges in some triangle $u,u',u''$. If $u\in D$, then point *p* is at distance at most $3/2\le \delta$ from point $u\in S_D$. If $u\notin D$, then vertex *u* is dominated by some vertex $v\in D$ with $\{u,v\}\in E_H$. Then in graph *G* the point $v\in S_D$ has an edge to all three points $u,u',u''$; hence *v* is at distance at most $3/2\le \delta$ from *p*.

Finally, consider a point *p* on one of the five edges between two triangles $u,u',u''$ and $v,v',v''$. By symmetry, we may assume that $d(u,p)\le 1/2$ holds. Since vertex *u* is dominated by some $w\in D$, we conclude $d(w,p)\le d(w,u)+d(u,p)\le 1+1/2\le \delta$. Hence *p* is $\delta$-covered by some $w\in S_D$. $\square$

### Lemma 4.4

If $\delta -{Cover}(G)\le k$, then graph *H* possesses a colorful dominating set.

### Proof

Consider a *k*-element $\delta$-cover $S\subseteq P(G)$. For every color class $V_i$, we define a corresponding point cloud $C_i\subseteq P(G)$ that contains (i) all points on the edges of the clique $V_i\cup \{x_i,y_i\}$ together with (ii) all points $p\in P(G)$ that satisfy $d(p,v)<1/2$ for some $v\in V_i$. Note that the $\delta$-cover *S* must contain at least one point from every cloud $C_i$: Otherwise, the midpoint $p(x_i,y_i,1/2)$ of the edge $\{x_i,y_i\}$ would not be $\delta$-covered by *S* (here we use that $\delta <2$). As there are *k* clouds and as clouds are disjoint, this yields that *S* contains precisely one point $c_i$ from every cloud $C_i$ and that *S* does not contain any further points.

Next we define $c^*_i$ as the point in $V_i$ that is closest to point $c_i$. In case $c_i$ is the midpoint of an edge in the clique $V_i$, then either end-vertex of that edge may be chosen as $c^*_i$. We claim that $\{c^*_1,\ldots ,c^*_k\}$ forms a colorful dominating set for *H*. As the midpoint $m_u=p(u',u'',1/2)$ of edge $\{u',u''\}$ in *G* is $\delta$-covered by *S*, there exists a covering point $s\in S$ with $d(m_u,s)\le \delta <2$. Since $m_u$ is at distance 3/2 from every vertex in $\Gamma ^+(u)$, we conclude $d(s,z)\le \delta -3/2<1/2$ must hold for some $z\in \Gamma ^+(u)$. This implies that *z* is one of the points $c^*_i$, exactly as we desired. $\square$

Lemmas 4.3 and 4.4 together yield the following summarizing statement.

### Lemma 4.5

For every fixed rational $\delta$ with $3/2\le \delta <2$, the $\delta$-covering problem with the solution size *k* as parameter is *W*[2]-hard. $\square$

The following two Lemmas 4.6 and 4.7 settle the remaining cases for the proof of Theorem 4.1.

### Lemma 4.6

For every integer $\ell \ge 2$ and for every rational $\delta$ with $\ell \le \delta <\ell +1/2$, the $\delta$-covering problem with the solution size *k* as parameter is *W*[2]-hard.

### Proof

We fpt-reduce from the classical *W*[2]-hard DOMINATING SET problem with solution size *k* as parameter: Given a graph $H=(V_H,E_H)$ and an integer *k*, decide whether *H* possesses a dominating set of size *k*. We construct the following instance $G=(V_G,E_G)$ of $\delta$-covering: The graph *G* results from graph *H* by attaching to every vertex $v\in V_H$ a simple path $P_v$ on $\ell -1$ edges. We claim that *H* has a dominating set *D* of size *k*, if and only if graph *G* allows a $\delta$-cover *S* of size *k*.

(Only if). Let *D* be a dominating vertex set of size *k* in *H*. We claim that the corresponding point set *D* forms a $\delta$-cover in graph *G*. Let $p\in P(G)$. If *p* lies on some edge $\{u,v\}\in E_H$, then *D* contains a vertex *z* with $d(z,v)\le 1$. We get $d(z,p)\le d(z,v)+d(v,p)\le 2\le \delta$, as desired. If *p* lies on the path $P_v$ attached to *v*, then *D* contains a vertex *z* with $d(z,v)\le 1$. We get $d(z,p)\le d(z,v)+d(v,p)\le 1+(\ell -1)\le \delta$, as desired.

(If). Let *S* be a $\delta$-cover of size *k* in *G*. We pick for every point $s\in S$ a closest point $s^*\in V_H$. If *s* is the midpoint of an edge in $V_H$, then either end-vertex may be chosen as $s^*$. We claim that the set $S^*:=\{s^*|s\in S\}$ forms a dominating set in graph *H*. Indeed, consider some vertex $v\in V_H$ and let $v'$ denote the vertex at the other end of the path $P_v$. The $\delta$-cover *S* contains some point *s* with $d(s,v')\le \delta <\ell +1/2$. If point *s* lies on $P_v$, then $s^*=v$ and *v* is dominated by $S^*$. If point *s* does not lie on $P_v$, then $d(v',v)=\ell -1$ implies $d(v,s)<3/2$; then $s^*\in \Gamma ^+(v)$ and *v* is dominated by $S^*$. $\square$

### Lemma 4.7

For every integer $\ell \ge 2$ and for every rational $\delta$ with $\ell +1/2\le \delta <\ell +1$, the $\delta$-covering problem with the solution size *k* as parameter is *W*[2]-hard.

### Proof

The proof is very similar to the proof of Lemma 4.6. We take an instance $H=(V_H,E_H)$ of DOMINATING SET, and we attach to every vertex $v\in V_H$ a gadget. The gadget consists of a path $P_v$ on $\ell -1$ edges that connects vertex *v* to vertex $v'$ and of two further vertices $v''$ and $v'''$ that together with $v'$ form a triangle $v'v''v'''$. It can be seen that *H* has a dominating set of size *k* if and only if *G* has a $\delta$-cover of size *k*. The details are analogous to those in the proof of Lemma 4.6 and hence are left to the reader. $\square$

Finally, we observe that the problems in Theorem 4.1 are not only *W*[2]-hard, but also contained in class *W*[2].

### Lemma 4.8

For every fixed rational $\delta$, the $\delta$-covering problem with the solution size *k* as parameter is contained in the complexity class *W*[2].

### Proof

Let $G=(V,E)$ be an input graph, and let $\delta =a/b$. Let $G'=(V',E')$ be the graph that results from *G* by subdividing every edge into 2*b* new edges. Let $G''=(V',E'')$ be the graph that results from $G'$ by connecting two vertices *u* and *v* by an edge, whenever they are at distance at most 2*a* in $G'$.

By Lemma 2.2, there exists an optimal $\delta$-cover *S* for *G* that is 2*b*-simple. By Lemma 2.1, this optimal $\delta$-cover *S* for *G* translates into a corresponding optimal 2*a*-cover $S'$ of $G'$, such that $S'\subseteq V'$ is a subset of the vertices. Then $S'$ is a dominating set for graph $G''$. This argument also works in the other direction, as any dominating set of size *k* in graph $G''$ corresponds to a $\delta$-cover of size *k* in graph *G*. All in all, this means that $\delta$-covering with the solution size as parameter is a special case of the dominating set with the solution size as parameter. The statement in the lemma now follows, since the dominating set problem is well-known to lie in *W*[2]; see for instance Downey & Fellows [3]. $\square$

## NP-hardness results

Theorem 4.1 trivially implies the NP-hardness of computing the $\delta$-covering number for all $\delta \ge 3/2$. The NP-hard cases in the range $\delta <3/2$ will be identified and settled on the following pages, and thereby yield the following theorem.

### Theorem 5.1

For every fixed positive rational $\delta$ that is not a unit fraction, it is NP-hard to compute the $\delta$-covering number of a graph.

Our main tools are the following two Lemmas 5.2 and 5.3. The first lemma is an immediate consequence of Lemma 2.1.

### Lemma 5.2

Let $\delta >0$ and let $c\ge 1$ be an integer. Suppose that the computation of the $\delta$-covering number is NP-hard. Then also the computation of the $(c\cdot \delta )$-covering number is NP-hard.

### Proof

By Lemma 2.1, subdividing every edge into *c* new edges yields a polynomial time reduction from $\delta$-covering to $(c\cdot \delta )$-covering. $\square$

### Lemma 5.3

Let $\ell$ and *r* be real numbers with $0\le \ell <r$. Suppose that for every rational $\delta$ with $\ell<\delta <r$, the computation of the $\delta$-covering number is NP-hard. Then the computation of the $\delta '$-covering number is also NP-hard for every rational $\delta '$ with

5 $$\begin{aligned} \frac{\ell }{2\ell +1}< \delta ' < \frac{r}{2r+1}. \end{aligned}$$

Furthermore, NP-hardness for the left boundary point $\delta =\ell$ implies NP-hardness for $\delta '=\ell /(2\ell +1)$, and in a symmetric way NP-hardness for the right boundary point $\delta =r$ implies NP-hardness for $\delta '=r/(2r+1)$.

### Proof

The function $f(x)=x/(2x+1)$ forms a bijection between the rational numbers in the open interval $(\ell ,r)$ and the rational numbers in the open interval $(\ell /(2\ell +1),r/(2r+1))$. Note that for $\delta =a/b$ we have $f(\delta )=a/(2a+b)$. The Shifting Theorem 3.1 yields that $\delta -{Cover}(G)=f(\delta )-{Cover}(G)-|E|$ for every graph $G=(V,E)$. Hence, computing the $\delta$-covering number is polynomial time reducible to computing the $f(\delta )$-covering number. $\square$

Now let us turn to the proof of Theorem 5.1, which is structured into three parts. The main trick is to alternately apply the tools developed in Lemmas 5.2 and 5.3.

In the first part of the proof, we define for every integer $j\ge 1$ a real number $\alpha _j=3^j/(3^j-1)$ and a half-open interval $A_j=[\alpha _{j+1},\alpha _j)$; furthermore we set $A_0=[3/2,\infty )$. Note that with the help of the function $f(x)=x/(2x+1)$ introduced in the proof of Lemma 5.3, we may equivalently write $\alpha _{j+1}=3f(\alpha _j)$. We prove by induction on $j\ge 0$ that for every rational $\delta \in A_j$, the $\delta$-covering number is NP-hard to compute. The anchor step with $j=0$ states NP-hardness for $\delta \in A_0=[3/2,\infty )$, and hence follows from Theorem 4.1. In the inductive step, we first apply Lemma 5.3 with $\ell =\alpha _j$ and $r=\alpha _{j-1}$ to interval $A_{j-1}$ and thus deduce hardness for all rational $\delta$ in the range $f(\alpha _j)\le \delta <f(\alpha _{j-1})$. Then we apply Lemma 5.2 with $c=3$ to deduce NP-hardness for all $\delta$ in the range $3f(\alpha _j)\le \delta <3f(\alpha _{j-1})$ and hence for all $\delta \in A_j$. This completes the inductive step. Since the numbers $\alpha _j$ form a decreasing sequence that converges to the limit point 1, the union of these intervals $A_j$ covers all numbers that are strictly larger than 1. Summarizing, in the first part we have established NP-hardness for all rational numbers $\delta >1$.

In the second part of the proof, we consider the open intervals $A'_j=(1/(2j+1),1/(2j))$ for $j\ge 0$. We prove by induction on $j\ge 0$ that for every rational $\delta \in A'_j$, the $\delta$-covering number is NP-hard to compute. The anchor step with $j=0$ claims NP-hardness for $\delta \in A'_0=(1,\infty )$, and hence follows from the above first part of our proof. In the inductive step, we apply Lemma 5.3 with $\ell =1/(2j+1)$ and $r=1/(2j)$, which yields $f(\ell )=1/(2j+3)$ and $f(r)=1/(2j+2)$. Thereby we lift NP-hardness from interval $A'_j$ to the next interval $A'_{j+1}$.

In the **third and last part of the proof**, we finally establish NP-hardness for all $\delta =a/b$ with $\gcd (a,b)=1$ and $a\ge 2$. Note that this settles all the positive rational numbers except for the unit fractions, and hence completes the proof of Theorem 5.1. The argument branches into two subcases. In the first subcase, we assume that the denominator *b* is odd. Since the integers 2*a* and *b* are relatively prime, there exists a positive integer *c* so that $bc\equiv 1\bmod {2a}$. In other words, there are positive integers *c* and *j* with $bc=1+2aj$. Then $2aj<bc$ implies $a/b<c/(2j)$, and $bc<a+2aj$ implies $a/b>c/(2j+1)$. We summarize these inequalities as

$$\begin{aligned} \frac{c}{2j+1}< \delta < \frac{c}{2j}. \end{aligned}$$

This means that $\delta /c\in A'_j$ (for the interval $A'_j$ as introduced in the second part of the proof), and that consequently $(\delta /c)$-covering is NP-hard. Then Lemma 5.2 implies that also $\delta$-covering is NP-hard.

In the second subcase, we assume that the denominator *b* is even with $b=2b'$; note that in this case the numerator satisfies $a\ge 3$. Since the integers *a* and $b'$ are relatively prime, there exists a positive integer *c* so that $b'c\equiv 1\bmod {a}$. In other words, there are positive integers *c* and *j* with $b'c=1+aj$. Then $aj<b'c$ implies $a/b<c/(2j)$, and $2b'c<a+2aj$ implies $a/b>c/(2j+1)$. Analogously to the first subcase, we conclude that $\delta /c\in A'_j$ and that $\delta$-covering is NP-hard.

This finally completes the proof of Theorem 5.1. It is instructive to find and to examine the places where the above argument breaks down for unit fractions $\delta =1/b$.

**Fig. 2:**
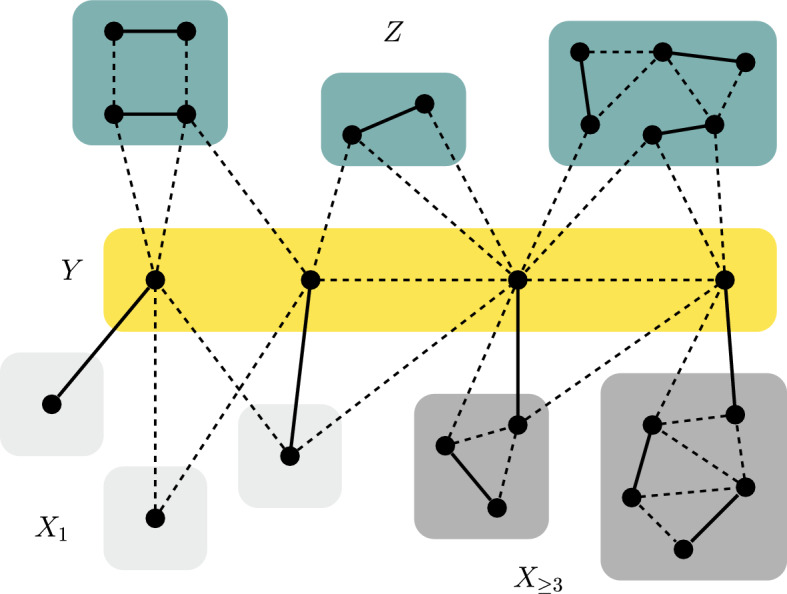
An illustration for the Edmonds-Gallai structure theorem. A maximum matching is shown in solid edges, while the non-matching edges are dashed

## The polynomial time result for 1-covering

In this section we derive a polynomial time algorithm for computing the 1-covering, which heavily relies on Edmonds-Gallai decompositions (Edmonds [5] and Gallai [7, 8]). The following theorem summarizes the Edmonds-Gallai result in a notation that is appropriate for our usage; Figure 2 gives an illustration.

### Theorem 6.1

(Edmonds [5] and Gallai [7, 8]) Let $G=(V,E)$ be a graph. The following decomposition of *V* into three sets *X*, *Y*, *Z* can be computed in polynomial time.

$$\begin{aligned} X= & {} \{v\in V\mid \text { there exists a maximum cardinality matching that misses }v\} \\ Y= & {} \{v\in V\mid v\notin X\text { and }v\text { is adjacent to some vertex in }X\} \\ Z= & {} V-(X\cup Y) \end{aligned}$$

Set *X* is the union of the odd-sized components of $G-Y$; every such odd-sized component is factor-critical. Set *Z* is the union of the even-sized components of $G-Y$. Every maximum cardinality matching in *G* induces a perfect matching on every component of *Z*, induces a near-perfect matching on every component of *X*, and matches the vertices in *Y* to vertices in different components of *X*. $\square$

Based on such an Edmonds-Gallai decomposition of $G=(V,E)$, we define the following three (pairwise disjoint) subgraphs of *G*:

- $G_0=(Z,E_0)$ is the subgraph of *G* induced by *Z*.
- Let $X_1$ contain the vertices that form components of size 1 in *X*, let $Y_1=\Gamma (X_1)$, and let $E_1$ denote the set of edges between $X_1$ and $Y_1$. We define $G_1=(X_1\cup Y_1,E_1)$ as the corresponding (not necessarily induced) bipartite subgraph of *G*.
- Let $X_{\ge 3}=X-X_1$ denote the vertices of *X* that belong to (odd-sized) components of size at least 3. We define $G_{\ge 3}=G[X_{\ge 3}]$ as the subgraph of *G* induced by $X_{\ge 3}$, and we let $c_{\ge 3}$ denote the number of components in $G_{\ge 3}$.

Now let us turn to 1-coverings. A 1-cover is in *canonical* form, if it entirely consists of vertices and of midpoints of edges. By Lemma 2.2 every graph possesses an optimal 1-cover in canonical form, and throughout this section, we will only consider (not necessarily optimal) 1-covers *S* in canonical form. Furthermore, in this section we will often simply write “cover” for “1-cover”. The *vicinity* $\text {vic}(v)$ of a vertex $v\in V$ consists of vertex *v* itself and of the midpoints of all edges incident to *v*.

### Lemma 6.2

Every 1-cover *S* satisfies

6 $$\begin{aligned} |S| \ge \nu (G_0) +\nu (G_{\ge 3})+c_{\ge 3}+\tau (G_1). \end{aligned}$$

### Proof

We define three sets $S_0=S\cap \text {vic}(Z)$, $S_{\ge 3}=S\cap \text {vic}(X_{\ge 3})$, and $S_1=S\cap P(G_1)$; we remind the reader that $P(G_1)$ denotes the continuum set of points on all the edges and vertices of $G_1$. We will now analyze the structure of these three sets and establish lower bounds on their sizes, which altogether will yield the lemma.

Our first lower bound is $|S_0|\ge \nu (G_0)$. To see this, consider a perfect matching $M\subseteq Z$ for graph $G_0$. Every vertex 1-covers at most one edge in *M*, and every midpoint of an edge 1-covers at most two half-edges in *M*. As $S_0$ is in canonical form and as $S_0$ must 1-cover all of *M*, this implies the desired bound $|S_0|\ge |M|=\nu (G_0)$.

Our second lower bound is $|S_{\ge 3}|\ge \nu (G_{\ge 3})+c_{\ge 3}$. To establish this bound, let us first analyze an arbitrary component *U* of graph $G_{\ge 3}$. Recall that |*U*| is odd and that *U* is factor-critical. We want to show that $|S_{\ge 3}\cap \text {vic}(U)|\ge (|U|+1)/2$, and for the sake of contradiction we assume the opposite $|S_{\ge 3}\cap \text {vic}(U)|\le (|U|-1)/2$.

- First consider the case where $S_{\ge 3}\cap \text {vic}(U)$ entirely consists of midpoints of edges. As every midpoint 1-covers only two vertices in *U*, there exists at least one vertex $u\in U$ that is not covered by the points in $S_{\ge 3}\cap \text {vic}(U)$, but by some outside vertex $y\in S\cap Y$. As *U* is connected and as $|U|\ge 3$, vertex *u* possesses a neighbor $u'\in U$. Then the points $p(u,u',\lambda )$ with $0<\lambda <1/2$ are not covered by *S*. That’s the desired contradiction.
- Next consider the case where $S_{\ge 3}\cap \text {vic}(U)$ contains some vertex $u\in U$. As *U* is factor-critical, the subgraph $G[U-u]$ contains a perfect matching *M* of size $(|U|-1)/2$. Similarly as before, we note that every vertex in $S_{\ge 3}\cap \text {vic}(U)$ covers at most one edge in *M* and that every midpoint in $S_{\ge 3}\cap \text {vic}(U)$ covers at most two half-edges of *M*. Since point *u* does not help in covering the edges in *M*, the $(|U|-1)/2$ points in $S_{\ge 3}\cap \text {vic}(U)$ cannot 1-cover all the edges in *M*. That’s the desired contradiction.

All in all, this yields the claimed inequality $|S_{\ge 3}\cap \text {vic}(U)|\ge (|U|+1)/2$ for component *U*. By adding these inequalities up over all the components *U* of $G_{\ge 3}$, we get

7 $$\begin{aligned} |S_{\ge 3}| \ge \sum _U \frac{1}{2}\,(|U|+1) = \sum _U \nu (U) +c_{\ge 3}= \nu (G_{\ge 3})+c_{\ge 3}. \end{aligned}$$

Here we used $\nu (U)=(|U|-1)/2$. Inequality (7) yields the desired lower bound on $|S_{\ge 3}|$.

Our third lower bound is $|S_1|\ge \tau (G_1)$. We define an auxiliary vertex set $C\subseteq X_1\cup Y_1$ as follows: Whenever $S_1$ contains the midpoint of an edge $\{x,y\}\in E_1$ with $x\in X_1$ and $y\in Y_1$, then set *C* contains the end-vertex *x* of that edge. Furthermore *C* contains all the vertices in $S_1$. Clearly $|C|\le |S_1|$.

Now consider an arbitrary edge $e=\{x,y\}\in E_1$ with $x\in X_1$ and $y\in Y_1$. The points $p(x,y,\lambda )$ with $0<\lambda <1/2$ are 1-covered by some point $s\in S$, and as *S* is in canonical form we actually have $s\in S_1$. We branch into three subcases: (i) If $s=x$ or $s=y$, then $s\in C$. (ii) If *s* is the midpoint of *e*, then $x\in C$. (iii) The only remaining possibility is that *s* is the midpoint of some other edge $e'\ne e$ incident to *x*, in which case $x\in C$ holds. As each of the three subcases leads to $x\in C$ or to $y\in C$, we conclude that *C* forms a vertex cover for the bipartite graph $G_1$. This yields the desired bound $|S_1|\ge |C|\ge \tau (G_1)$.

Finally, let us put things together. It is easily seen that the three sets $S_0,S_{\ge 3},S_1$ are pairwise disjoint. This implies $|S|\ge |S_0|+|S_{\ge 3}|+|S_1|$, which in combination with our three lower bounds then yields the inequality in (6). $\square$

Our next goal is to construct in polynomial time a 1-cover $S^*$ whose size matches the bound stated in Lemma 6.2, and which therefore is an optimal 1-cover. We start by computing a maximum cardinality matching $M\subseteq E$ for graph *G* and a minimum vertex cover $C\subseteq X_1\cup Y_1$ for the bipartite subgraph $G_1$. The set $S^*$ is then defined as follows:

(i) $S^*$ contains the midpoint of every edge in matching *M* that does not belong to the edges between *X* and *Y*.
(ii) $S^*$ contains every vertex $x\in X_{\ge 3}$ that is not saturated by *M*, and $S^*$ contains the midpoint of every edge $\{x,y\}\in M$ with $x\in X_{\ge 3}$ and $y\in Y$.
(iii) $S^*$ contains every vertex in $C\cap Y_1$, and furthermore contains every vertex in $C\cap X_1$ that is not saturated by *M*. If $x\in C\cap X_1$ is saturated by *M*, then $S^*$ contains the midpoint of the matching edge incident to *x*.

Note that (i) contributes $\nu (G_0)+\nu (G_{\ge 3})$ points to $S^*$, that (ii) contributes $c_{\ge 3}$ points, and that (iii) contributes $\tau (G_1)$ points. Hence the size of $S^*$ indeed matches the lower bound in (6). It remains to show that $S^*$ is a 1-cover for *G*.

- As *M* induces a perfect matching on *Z*, the set $S^*$ contains for every $z\in Z$ the midpoint of some incident edge. Hence every closed ball $B^+(z,1/2)$ is 1-covered by $S^*$.
- Every vertex $y\in Y$ is saturated by the matching *M*. If $y\in C\cap Y_1$ then $y\in S^*$, and if $y\notin C\cap Y_1$ then $S^*$ contains the midpoint of the matching edge incident to *y*. In either case the ball $B^+(y,1/2)$ is 1-covered by $S^*$.
- Let $x\in X_1$. If $x\in C$, then $S^*$ does either contain *x* itself or does contain the midpoint of an incident matching edge; in either case the ball $B^+(x,1/2)$ is 1-covered by $S^*$. If $x\notin C$, then every vertex $y\in \Gamma (x)$ lies in $C\cap Y_1$; in this case $S^*$ contains every neighbor $y\in \Gamma (x)$ so that the ball $B^+(x,1)$ is 1-covered by $S^*$.
- If a vertex $x\in X_{\ge 3}$ is saturated by *M*, then $S^*$ contains the midpoint of the incident matching edge. If $x\in X_{\ge 3}$ is not saturated by *M*, then by construction $x\in S^*$. In either case the ball $B^+(x,1/2)$ is 1-covered by $S^*$.

All in all, we have shown that for every vertex $v\in V$ the entire ball $B^+(v,1/2)$ is 1-covered by $S^*$. Hence all the edges in *G* are covered, and $S^*$ indeed is a 1-cover for *G*.

### Theorem 6.3

For every unit fraction $\delta$, the $\delta$-covering problem is solvable in polynomial time.

### Proof

The above discussion yields the polynomial time algorithm for $\delta =1$, and Lemma 2.1 extends this result to all unit fractions $\delta$. $\square$

## The fixed parameter tractable cases

Throughout this section, we consider some fixed rational number $\delta <3/2$ and some fixed integer *k*. We will develop an fpt-algorithm with parameter *k* for deciding whether an input graph *G* satisfies $\delta -{Cover}(G)\le k$, that is, a decision algorithm whose running time is bounded by some computable function *f*(*k*) and by some polynomial function in the instance size |*G*|.

In a preliminary step, we consider a vertex *v* that is incident to $\ell \le 4k$ edges. We denote by $g(\ell ,\delta )$ the minimum size of a $\delta$-cover for the open ball $B^-(v,1-\delta )$. Note that $g(\ell ,\delta )=0$ whenever $\delta \ge 1$. Note furthermore that the value $g(\ell ,\delta )$ can be computed in constant time, as the number $\ell$ is bounded in terms of the parameter ($\ell \le 4k$) and as $\delta$ does not depend on the input graph *G*. Now let us start working towards the fpt-algorithm.

### Lemma 7.1

For $\delta <3/2$, every graph $G=(V,E)$ satisfies $\delta -{Cover}(G)\ge \nu (G)/2$.

### Proof

Let $M\subseteq E$ be a maximum size matching in *G*, and let $S\subseteq P(G)$ be a minimum size $\delta$-cover. Then any point $p\in S$ will $\delta$-cover at most two of the midpoints of edges in *M*. (This is the only place in this section where we exploit the condition $\delta <3/2$.)

$$\square $$

Our fpt-algorithm first computes a maximum cardinality matching *M* for the input graph *G*. If $|M|>2k$ then the algorithm outputs NO and stops; this step is justified by Lemma 7.1, as $\delta -{Cover}(G)>k$ implies a negative answer to the decision problem. Hence from now on we will assume $|M|\le 2k$. Then the end-vertices of the edges in *M* form a vertex cover *C* of size $|C|\le 4k$ and the vertices in $I=V-C$ form an independent set. For $T\subseteq C$, we let $I_T$ denote the set of all vertices $v\in I$ with $\Gamma (v)=T$.

### Lemma 7.2

Let *S* be an optimal $\delta$-cover for the graph $G=(V,E)$ and let $T\subseteq C$. Then in the set $I_T$ there are at most |*T*| vertices *v* for which $S\cap B^-(v,1)$ contains at least $g(|T|,\delta )+1$ points.

### Proof

Suppose otherwise. Then there are at least $|T|+1$ vertices *v* in $I_T$ for which the ball $B^-(v,1)$ contains at least $g(|T|,\delta )+1$ points from *S*. For all other vertices $v\in I_T$, the ball $B^-(v,1)$ contains at least $g(|T|,\delta )$ points from *S*. All in all, this yields that *S* contains at least $|I_T|\,g(|T|,\delta )+|T|+1$ points from these open balls around vertices in $I_T$. We remove all these points from set *S* and replace them by the following points:

- We add all the points in *T* to *S*.
- For every vertex $v\in I_T$, we add $g(|T|,\delta )$ points to *S* that $\delta$-cover $B^-(v,1-\delta )$.

The resulting point set is again a $\delta$-cover for *G*, but does contain strictly fewer points than the optimal $\delta$-cover *S*. This yields the desired contradiction. $\square$

In the following paragraphs, we investigate a subset $T\subseteq C$ that satisfies $|I_T|\ge 2|T|+1$. Recall that $I_T$ is an independent set and that every vertex $v\in I_T$ satisfies $\Gamma (v)=T$. We denote by $\mathcal{B}_T$ the union of all the open balls $B^-(v,1)$ with $v\in I_T$. Now consider some fixed optimal $\delta$-cover *S* for the underlying graph *G*.

- For every vertex $t\in T$, consider a point $s_t\in S\cap \mathcal{B}_T$ that minimizes the distance $d(s_t,t)$. Whenever some point $p\notin \mathcal{B}_T$ is $\delta$-covered by a point from $S\cap \mathcal{B}_T$, then this point *p* will also be covered by one of the points $s_t$ with $t\in T$. If $s_t\in B^-(v,1)$, then we say that the corresponding vertex *v* is *busy*. There are at most |*T*| busy vertices in $I_T$.
- A vertex *v* in $I_T$ is *heavy*, if $B^-(v,1)$ contains at least $g(|T|,\delta )+1$ points from *S*. By Lemma 7.2, there are at most |*T*| heavy vertices in $I_T$.

Since $|I_T|\ge 2|T|+1$, there exists a vertex $w\in I_T$ that is neither busy nor heavy. What can we say about the points in the set $S\cap B^-(w,1)$? Since vertex *w* is not heavy, $S\cap B^-(w,1)$ must contain exactly $g(|T|,\delta )$ points. Since vertex *w* is not busy, the points in $S\cap B^-(w,1)$ are not required for $\delta$-covering the points outside of $\mathcal{B}_T$. In other words, the only duty of these points in $S\cap B^-(w,1)$ is to cover the open ball $B^-(w,1)$. This observation allows us to eliminate vertex *w*, as it is dispensable and only imposes minor constraints on the structure of a $\delta$-cover. We define a new instance by removing vertex *w* (together with its |*T*| incident edges) from *G* and by simultaneously decreasing the parameter *k* by the value $g(|T|,\delta )$.

### Lemma 7.3

The new instance $G-w$ and $k-g(|T|,\delta )$ is a YES-instance of $\delta$-covering, if and only if the old instance *G* and *k* is a YES-instance of $\delta$-covering.

### Proof

For the (if) part, assume that *G* has a $\delta$-cover of size at most *k*. Let *S* be an optimal $\delta$-cover with $|S|\le k$. We pick a vertex $w\in I_T$ that is neither busy nor heavy, and remove the $g(|T|,\delta )$ points in $S\cap B^-(w,1)$ from *S*. This yields a $\delta$-cover of size $k-g(|T|,\delta )$ for $G-w$.

For the (only if) part, assume that the graph $G-w$ has a $\delta$-cover $S'$ of size at most $k-g(|T|,\delta )$. We pick an arbitrary vertex $u\in I_T$ with $u\ne w$, and we use a clone of the set $S'\cap B^-(u,1)$ to cover $B^-(w,1)$. This extends $S'$ to a $\delta$-cover of *G* of size at most *k*. $\square$

The above discussion yields the following reduction rule: *“Whenever some subset* $T\subseteq C$ *satisfies* $|I_T|\ge 2|T|+1$, *then we may remove a vertex* $w\in I_T$ *from* *G* *and decrease the parameter* *k* *by* $g(|T|,\delta )$*.”* We apply this reduction rule over and over again, until no further reductions are possible. At termination, the vertex set of the residual graph will consist of a vertex cover *C* together with the shrunken independent sets $I_T$ that now satisfy $|I_T|\le 2|T|\le 8k$. Hence the residual graph has at most $4k(1+2^{4k+1})$ vertices. As this size is bounded by a function in the parameter *k* and as the parameter is not part of the input, the resulting instance of $\delta$-covering can be solved within a time complexity that only depends on the parameter. Since the reduction rule can easily be implemented in polynomial time, we formulate the following summarizing theorem.

### Theorem 7.4

For every fixed rational $\delta$ with $\delta <3/2$, the $\delta$-covering problem with the solution size *k* as parameter allows an fpt-algorithm. $\square$

## Conclusion

We have fully analyzed the computational complexity of $\delta$-covering for positive rational values of $\delta$. The problem is easy if $\delta$ is a unit fraction, and otherwise it is NP-complete. We have also fully analyzed the parametrized complexity of $\delta$-covering for positive rational values of $\delta$. The problem is in FPT for all $\delta <3/2$, and *W*[2]-complete for all $\delta \ge 3/2$.

As an open problem, we suggest to study the computational complexity of the decision problem with *algebraic* real $\delta$. These decision problems are contained in NP, and we conjecture that they are NP-hard for all algebraic values $\delta$ that are not unit fractions.
